# Alkylation of DNA In Vivo: Development of Analytical Methodology for Trace Quantitative Analysis

**DOI:** 10.6028/jres.093.101

**Published:** 1988-06-01

**Authors:** R. G. Cooks, J. R. O’Lear, C.-j. Chang

**Affiliations:** Departments of Chemistry and Medicinal Chemistry, Purdue University, West Lafayette, IN 47907

## 1. Abstract

The application of tandem MS techniques to the determination of the site and extent of alkylation of DNA by chemical carcinogens is illustrated. It is shown that it is possible to i) separate many methyldeoxyribonucleosides and the common deoxyribonucleosides in a single LC run, ii) detect and quantify pure methyldeoxyribonucleosides at the 10^−14^ mole level by desorption chemical ionization tandem mass spectrometry, and iii) quantify the major methylated nucleosides resulting from treatment of calf thymus DNA or hamster V79 cells with methylnitrosourea (MeNU) or methylmethanesulfonate (MeMS). The ultimate aim is to use *in vivo* experiments to correlate mutagenicity and cytotoxicity of the alkylating agents with the type and distribution of the alkylated adducts and with their metabolic half-lives (metabolic persistence/repair) in cell cultures.

## 2. Significance

Alkylating agents [1] exhibit pronounced biological activity. Many are mutagenic in various genetic systems [1–3] and more than a dozen are being used clinically as anticancer drugs [4,5]. Reactions *in vitro* between alkylating agents and nucleic acids and their biological consequences in terms of transcription and translation activities have been reviewed [3,6]. Almost all nitrogen and oxygen atoms of DNA bases can be alkylated. In general, nitrosoureas (strong mutagens) yield a higher percentage of oxygen-alkylated products, suggesting that the cell transformation activity of alkylating agents may be related to the degree of oxygen-alkylation. In recent years, much interest has focused on O^6^-alkylation of guanine because this modification can result in base mispairing [1,7–9]. Increasing attention has also been directed toward the formation and metabolic persistence of O^4^-alkylthymine [10–12]. The formation of O^4^-methylthymidine, 3-methylthymidine, 3-methyldeoxycytidine, and 1-methyldeoxyadenosine in V79 cells by MeMS or MeNU has been demonstrated in our study. Further studies on the extents of their formation and metabolic persistence of these modifications upon exposure to various alkylating agents at different dosages are essential for an understanding of their differential toxicity and mutagenicity at the molecular level.

## 3. Analytical Methodology

### 3.1 Overall Approach

**Figure 1:**
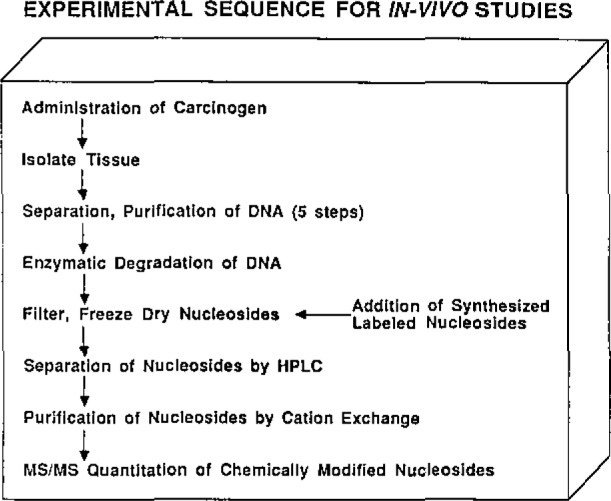
The analytical protocol.

Studies of DNA alkylation *in vivo* challenge analytical chemistry by requiring qualitative and quantitative analysis at high sensitivity for closely related compounds, some of which are involatile and thermally unstable and all of which occur in particularly complex matrices. Fortunately, recent progress in liquid chromatography and mass spectrometry offers capabilities to match these requirements. Figure 1 illustrates the protocol adopted.

Implicit in this methodology is the requirement for the synthesis of deuterium-labeled nucleosides. The methylated nucleosides examined in this investigation were 3-methyldeoxycytidine (m^3^dc), 1-methyldeoxyadenosine (m^1^dA), 7-methyldeoxyguanosine (m^7^dG), 1-methyldeoxyguanosine (m^1^dG), 3-methylthymidine (m^3^T), O^4^-methylthymidine (m^4^T), O^2^-methylthymidine (m^2^T), 3-methyladenine (m^3^Ade) and O^6^-methyldeoxyguanosine (m^6^dG). All compounds were synthesized as the trideuteromethyl nucleosides, and as the unlabeled methyl compounds.

The task of separation of isomeric alkylated nucleosides utilized high pressure liquid chromatography. Satisfactory separations of the various methylated compounds in reasonable times can be achieved by a procedure which uses ion-pair reverse-phase chromatography [13,14]. The technique of tandem mass spectrometry (MS/MS) [15,16] also provides separation capabilities as well as specific, sensitive detection and very rapid sample throughput. Desorption ionization, with tandem mass spectrometry, is the key to the application of mass spectrometry to this problem. The desorption ionization procedures [17,18] have had considerable influence on the application of mass spectrometry to the biological sciences [19], increasingly through the simultaneous use of MS/MS [20,21]. The particular form of desorption ionization used here, desorption chemical ionization (DCI), employs rapid heating of a sample held in a chemical ionization plasma [22,23].

Analysis of the pattern of alkylation of Chinese hamster V79 cells employs a triple quadrupole instrument for desorption chemical ionization followed by tandem mass spectrometry. Quantification is based on daughter spectra of the modified nucleosides for the major methylation products and on selected reaction monitoring spectra of the minor components. The rapidity and specificity of MS/MS analysis are important advantages which, coupled with its ultra-high sensitivity, permit the detection and quantification of 0.1–10 ng of individual modified nucleosides. It should be feasible for us to measure the alkylated nucleosides in the ranges of 0.1 to 10 picomoles resulting from *in vivo* alkylation of DNA with chemical carcinogens.

### 3.2 HPLC Separation

To allow the accurate determination by chemical ionization of the amounts of methylated nucleosides present relative to deuterium-labeled internal references, it was necessary to develop an HPLC system capable of separating these nucleosides. Although absolute resolution of each peak in the chromatographic system was not rigorously required, an initial objective was the complete resolution of all structural isomers being analyzed. The separation achieved with the high-pressure liquid chromatographic system (CH_3_CN/H_2_O; 50 mmol/L HCOONH_4_; 3 mmol/L NEt_4_Cl; C-18 column) is excellent. The deoxyguanosine isomers, m^7^dG, m^1^dG and m^6^dG; deoxyadenosine isomers, m^1^dA and m^6^dA; deoxycytidine isomers, m^3^dC and m^5^dC, as well as the thymidine isomers m^2^T, m^3^T, and m^4^T are all completely separated.

### 3.3 Ionization Method

While DCI is not capable of ionizing the larger biomolecules which can be examined by ^252^Cf-plasma desorption [24–26], secondary ion mass spectrometry (SIMS) [27,28] or fast atom bombardment (FAB) [29–32], it is a superior technique in this work since the absence of a matrix results in much lower detection limits than are achievable by the other methods. The suitability of this relatively simple ionization method for analysis of nucleosides has been recognized by others [33]. Experiments with solid (static) SIMS and laser desorption were successful, but failed to exhibit the low detection limits of DCI.

### 3.4 Tandem Mass Spectrometry: Daughter Ion Mode

**Figure 2:**
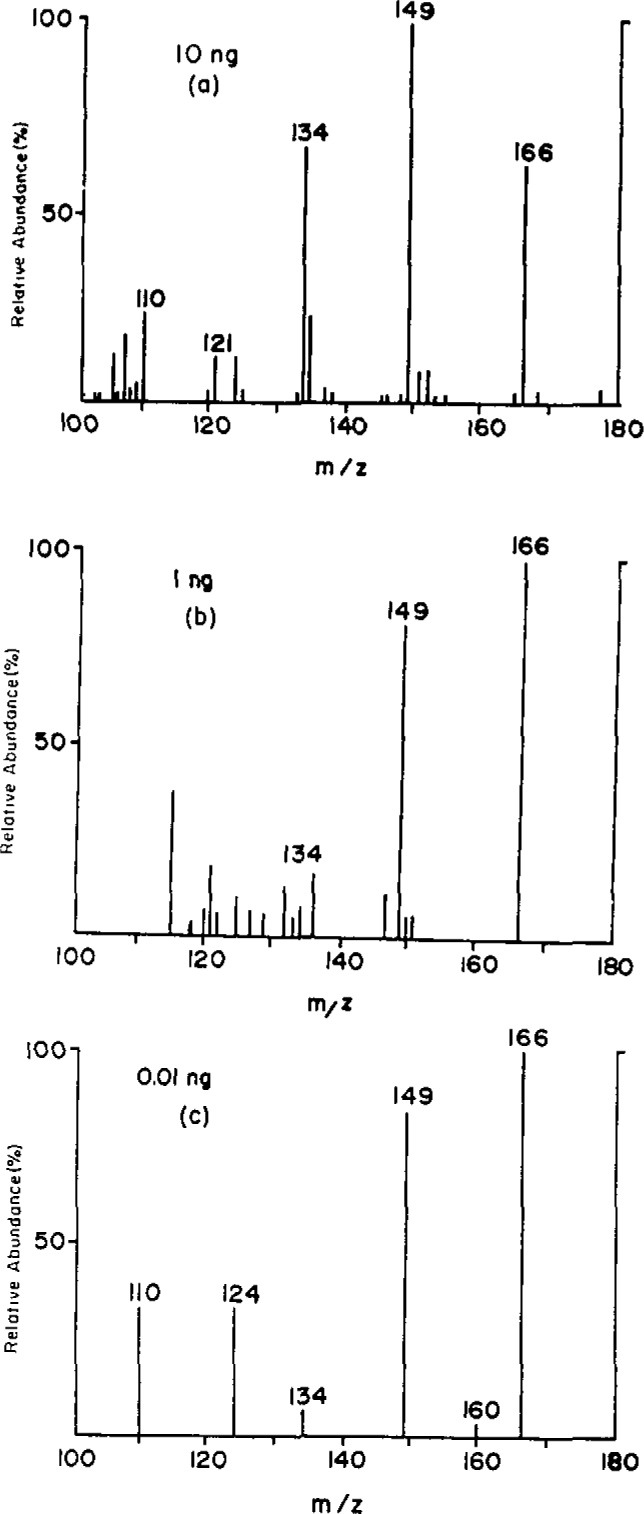
MS/MS data for O^6^-methyldeoxyguanosine (a) daughter spectrum of (M+H)^+^, *m/z* 166, isobutane CI, multiple Ar collisions, triple quadrupole 20 eV collision energy; (b) daughter spectrum, ammonia CI; (c) selected reaction monitoring, reactant ion 166.

The biologically significant minor alkylation product, m^6^dG, could not be quantified by singlestage MS analysis [34] because of the poor signal-to-noise in the mass spectrum. MS/MS has therefore been used to increase specificity and sensitivity [13]. Figure 2a shows the daughter spectrum of *m/z* 166 for 10 ng (36 pmol) of m^6^dG. The sensitivity is further improved by using ammonia instead of isobutane as ionization gas (fig. 2b). These data indicate a striking enhancement in specificity and sensitivity for tandem mass spectrometry over single-stage mass spectrometry.

### 3.5 Tandem Mass Spectrometry: Selected Reaction Monitoring Mode

The sensitivity of the MS/MS experiment can be enhanced even more by a selected reaction monitoring technique. Figure 2c shows the selected reaction monitoring spectrum of 0.01 nanogram (35 femtomole) of m^6^dG. This technique has less specificity than scanning the entire tandem mass spectrum because only a few peaks are monitored; however, it has the important advantage of remarkably increased sensitivity due to the fact that all of the analysis time is spent monitoring reactions of interest. This advantage has also been demonstrated in determinations of m^7^dG [34]. The procedure has the further advantage that the background noise level can be monitored by selecting for examination fragment ions which do not arise from the analyte.

### 3.6 Quantitative Analysis by Tandem Mass Spectrometry

Quantitation of pure methylnucleosides with tandem mass spectrometry by using internal standards labeled with deuterium yields excellent linearity in the low ng level [35].

## 4. Results of DCI MS/MS Analysis

### 4.1 Tandem Mass Spectrometrie Analysis of Methylation of Calf Thymus DNA

**Figure 3:**
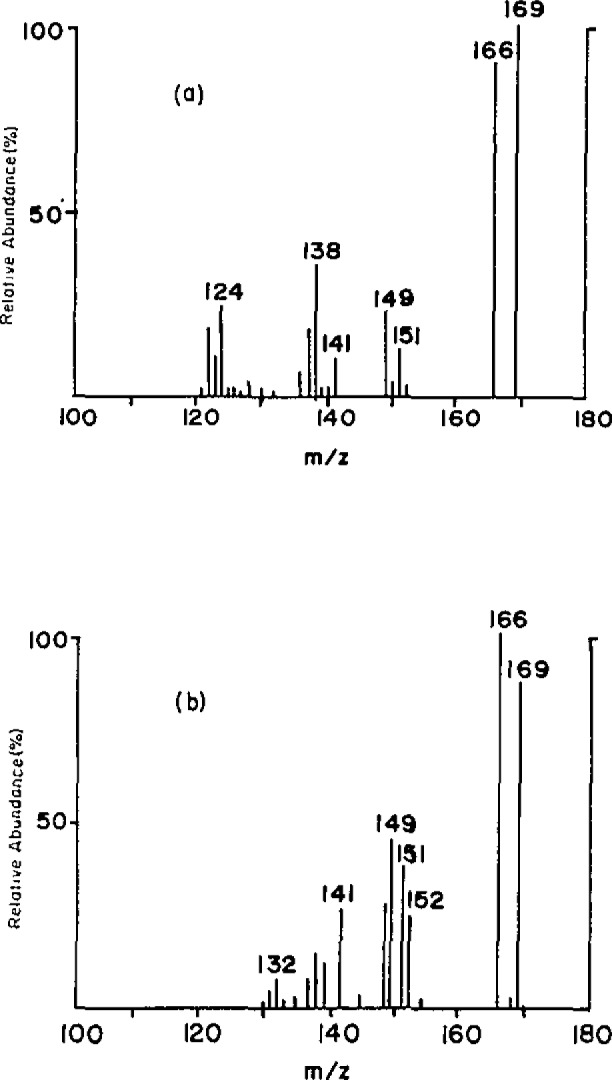
Daughter spectra (Ar, 20 eV, multiple collisions) for quantification of (a) m^7^dG and (b) m^6^dG using DCI selecting the protonated molecule (*m/z* 166) and internal standard (*m*/*z*) 169) in each case.

Calf thymus DNA was methylated with methyl methanesulfonate [molar ratio (MeMS/DNA-P)=25] at pH 7 for 12 hours. Deuterium labeled 7-methyldeoxyguanosine and O^6^-methyldeoxyguanosine were added to the methylated DNA as internal references, subjected to standard enzymatic degradation into nucleosides, and then separated by HPLC. The desorption chemical ionization daughter ion spectra of the corresponding HPLC fractions are shown in figure 3. The quantitative determination is readily made on the basis of the peak ratio of the CH_3_-analyte and the CD_3_-reference compound and the known amount of the reference compound. The results are shown in table 1, the last column of which is a comparison with the amounts of the methylated nucleosides estimated from other experiments.

### 4.2 Alternative Approach for Tandem Mass Spectrometric Analysis of Nucleosides

**Figure 4:**
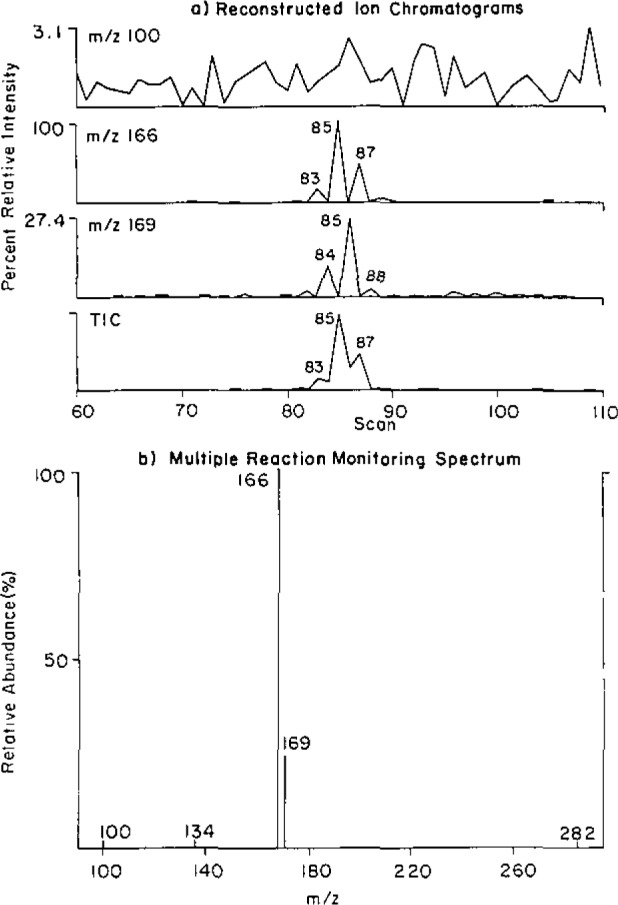
Multiple reaction monitoring experiment to quantitate m^6^dG (M+H)^+^ = 166 using the d_3_ analog (10 ng) (M+H)^+^ = 169 in a methylated calf thymus DNA sample. Ionization by DCI, 100 °C/s; fragmentation by 20 eV multiple collisions with argon (a) shows the individual ion chromatograms; (b) shows the multiple reaction monitoring data.

The DCI/MS/MS experiment using multiple reaction monitoring or daughter scans to characterize the protonated base is a considerable improvement over the earlier CI single-stage MS experiments. However, for *quantitation* of the *minor* products of methylation, the method suffers from unacceptably high background signals. An alternative approach for determining methyldeoxyribonucleosides is to measure the nucleoside molecular ion peak. The daughter ion MS/MS spectrum of the pseudomolecular ion (M + H^+^ = 282) of O^6^-methyldeoxyguanosine (1 ng) shows an intense peak at *m/z* 166, corresponding to the fragment of O^6^-methylguanine. The corresponding spectrum of the solvent blank displays a very weak peak at *m/z* 166 despite the fact that the mass spectrum includes a strong 282 peak. The specificity of MS/MS therefore avoids contributions to the signal from the 282 background. We validated the quantification of O^6^-methyldeoxyguanosine by mixing equal amounts of deuterated standard and analyte with enzymatically degraded nonmethylated DNA at the 4 ng level. After going through the analysis scheme the standard deviation was less than 0.2 ng. Therefore analysis at the nucleoside level appears to overcome the background interference problem. Application of this methodology gave results which are illustrated for m^6^dG produced by MeNU action on calf thymus DNA (fig. 4). Note that the selected reaction monitoring experiment rather than the full daughter scan will be used to further lower detection limits.

In order to evaluate reproducibility, a sample resulting from methylation of calf thymus DNA with methylnitrosourea (pH 7, 3 hours, MeNU/DNA-P=5) was divided into three portions and separately analyzed (table 2). These results show reasonable precision although consideration of the data collection algorithm reveals a potential source of error. Since the reactions which characterize the labeled standard and the analyte are interrogated alternatively during the short period (2–3 s) that the sample is evaporated, discrimination against one or another signal may occur, depending on the relationship between the evaporation profile of the sample and the points at which data are acquired by the instrument. Consideration of this factor [35] should result in much better reproducibility.

## 5. Conclusion

This project is helping in answering, at the molecular level, questions on the extent and types of reaction of alkylating agents with DNA, and how the *in vivo* persistence of these products correlates with relative cytotoxicity and mutagenicity. It is also providing powerful analytical techniques for the study of the differential toxicity of anticancer alkylating agents in terms of their interaction with cellular DNA. Screening for exposure to alkylating chemicals is a long-term objective for which, with further refinement of the methodology proposed here, the LC/MS/MS methods must be important candidates.

## Figures and Tables

**Table 1:** Quantitative MS/MS analysis of methyldeoxyguanosines in calf thymus DNA treated with methylmethanesulfonate (analysis at base level)

Nucleoside	[^2^H_3_] methyl nucleoside reference (nmol)	Relative ratio of [^1^H_3_] methyl [^2^H_3_] methyl nucleosides	[^1^H_3_] methyl nucleoside formed (nmol)	[^1^H_3_] methyl nucleoside estimated (nmol)
m^7^dG	1.9	0.91	1.7	1.9^a^
m^6^dG	3.9×10^−3^	1.15	4.5×10^−3^	3.9×10^−3^^b^

a The amount of m^7^dG formed is estimated from HPLC results using thymidine as an internal reference.

b The amount of m^6^dG formed is roughly estimated from the earlier single-stage mass spectrometric analysis containing high experimental error due to the poor signal-to-noise ratio.

**Table 2:** Quantitative DCI MS/MS analysis of O^6^-methyldeoxyguansine in calf thymus DNA treated with methylnitrosourea (analysis at nucleoside level)

[^2^H_3_] nucleoside reference (mol)	[^1^H_3_] methyl nucleoside formed (mol) uncorrected
8.5×10^−12^	2.8×10^−11^
1.1×10^−11^	3.8×10^−11^
1.1×10^−11^	5.4×10^−11^
